# Exploring the Link between Chronic Kidney Disease and Parkinson’s Disease: Insights from a Longitudinal Study Using a National Health Screening Cohort

**DOI:** 10.3390/nu15143205

**Published:** 2023-07-19

**Authors:** Mi Jung Kwon, Jwa-Kyung Kim, Ji Hee Kim, Joo-Hee Kim, Min-Jeong Kim, Nan Young Kim, Hyo Geun Choi, Eun Soo Kim

**Affiliations:** 1 Department of Pathology, Hallym University Sacred Heart Hospital, Hallym University College of Medicine, Anyang 14068, Republic of Korea; mulank@hanmail.net; 2 Division of Nephrology, Department of Internal Medicine, Hallym University Sacred Heart Hospital, Hallym University College of Medicine, Anyang 14068, Republic of Korea; kjk816@hallym.or.kr; 3 Department of Neurosurgery, Hallym University Sacred Heart Hospital, Hallym University College of Medicine, Anyang 14068, Republic of Korea; kimjihee.ns@gmail.com; 4 Department of Medicine, Division of Pulmonary, Allergy, and Critical Care Medicine, Hallym University Sacred Heart Hospital, Hallym University College of Medicine, Anyang 14068, Republic of Korea; luxjhee@gmail.com; 5 Department of Radiology, Hallym University Sacred Heart Hospital, Hallym University College of Medicine, Anyang 14068, Republic of Korea; drkmj@hallym.or.kr; 6 Hallym Institute of Translational Genomics and Bioinformatics, Hallym University Medical Center, Anyang 14068, Republic of Korea; honeyny78@gmail.com; 7 Suseo Seoul E.N.T. Clinic and MD Analytics, 10, Bamgogae-ro 1-gil, Gangnam-gu, Seoul 06349, Republic of Korea; mdanalytics@naver.com

**Keywords:** chronic kidney disease, Parkinson’s disease, longitudinal follow-up study, nationwide health insurance research database

## Abstract

Chronic kidney disease (CKD) and Parkinson’s disease (PD) are common illnesses found in the geriatric population. A potential link between CKD and PD emergence has been hypothesized; however, existing conclusions are disputed. In this longitudinal research, we analyzed data acquired from the Korean National Health Insurance Service-Health Screening Cohort. The dataset comprised the health information of 16,559 individuals clinically diagnosed with CKD and 66,236 control subjects of comparable ages, all aged ≥40 years. These subjects participated in health examinations from 2002 to 2019. To assess the correlation between CKD and PD, we employed overlap-weighted Cox proportional hazard regression models. The unadjusted, crude hazard ratio for PD was greater in the CKD group than in the control group (crude hazard ration (HR) 1.20; 95% confidence interval (CI) = 1.04–1.39; *p* = 0.011). However, the Cox proportional hazard regression analysis, incorporating propensity score overlap weighting, revealed no significant discrepancy after considering confounding variables such as demographic factors, socio-economic status, lifestyle, and concurrent health conditions (adjusted HR (aHR), 1.09; 95% CI = 0.97–1.22; *p* = 0.147). Subgroup analyses showed a higher probability of PD development among certain CKD individuals, including those who resided in rural areas (aHR, 1.19; 95% CI = 1.03–1.37; *p* = 0.022), maintained a normal weight (aHR, 1.29; 95% CI = 1.08–1.56; *p* = 0.006), or had fasting blood glucose levels ≥100 mg/dL (aHR, 1.18; 95% CI = 1.00–1.39; *p* = 0.046). Therefore, these clinical or environmental factors may influence the incidence of PD in CKD patients. In conclusion, our results suggest that the general CKD population may not exhibit a greater propensity for PD than their non-CKD counterparts. However, this might be contingent upon specific lifestyle and comorbid conditions. Thus, certain lifestyle alterations could be crucial in mitigating the potential manifestation of PD in patients diagnosed with CKD.

## 1. Introduction

Chronic kidney disease (CKD) and Parkinson’s disease (PD) are frequently occurring long-term illnesses which are prevalent among the aging population [1,2]. CKD is a significant health issue worldwide and impacts approximately 10–13 percent of the overall population in Korea [1,3]. Notably, the occurrence of CKD increases to approximately 40% among individuals aged ≥60 years [4]. It is defined as enduring abnormalities in kidney structure or functionality that extend beyond a period of 3 months [5]. Without proper treatment, CKD may deteriorate to a stage necessitating dialysis or kidney transplant [6]. Furthermore, CKD significantly escalates the risk for cardiovascular incidents and raises the likelihood of mortality from all causes [6]. In fact, from 1990 to 2017, the worldwide mortality rate due to CKD in all age categories surged by 41.5% [7].

In addition, PD, which is the second-most prevalent neurodegenerative disease, exhibits its top incidence rates among individuals who are over 60 years of age [8]. PD is clinically typified by motor symptoms such as resting tremors, bradykinesia (movement sluggishness), rigidity, and postural instability [9]. Pathologically, it is marked by α-synucleinopathy and the selective loss of dopaminergic neurons within the nigrostriatal pathway in the brain [9]. In Korea, the prevalence of PD, adjusted for age and sex, has escalated from 115.9 to 139.8 cases per 100,000 individuals between 2010 and 2015, with an annual incidence of 23.9 cases per 100,000 population [8]. This growth rate surpasses those observed in other nations; for example, the annual incidence rose by a factor of 1.17 in the USA between the periods 1986–1995 and 1996–2005 [10]. In Taiwan, the increase was by a factor of 0.97 from 2005 to 2011 [11], whereas in the United Kingdom it was 0.60 from 1999 to 2009 [12], and in the Netherlands, it was 0.36 from the 1990s to the 2000s [13]. Given the demographic shift towards an older population and the evolving lifestyles in Korea [14], CKD and PD have surfaced as considerable public health issues. These conditions impose a substantial economic and health strain on society.

Accumulating evidence indicates a potential connection between CKD and PD [15,16,17,18,19,20]. For example, initial case reports and case series studies sporadically reported PD incidence in CKD patients, a majority of whom were of Asian heritage [15,16]. Subsequent cohort studies (two Taiwanese and one Korean) have advocated that CKD could heighten the risk of PD or parkinsonism in CKD patients compared to control groups [17,18,19]. Additionally, a human disease network analysis proposed CKD as a potent hazard element for PD, presenting an odds ratio of 8.5 [20]. This association may raise concerns about the heightened comorbidity risk of PD in elderly individuals with CKD. These findings may lend credence to the hypothesis of bidirectional interactions between the kidneys and the nervous system, as demonstrated in both in vitro and in vivo research [21,22]. It is known that persistent renal injury and diminished renal function can adversely impact the function and structure of various organ systems, including the brain, gut, lungs, heart, and immune system [23,24]. Therefore, it is plausible that CKD and PD may share common risk factors and underlying mechanisms. These common risk factors may include advanced age, specific genetic predispositions, exposure to certain environmental toxins, chronic inflammation, cardiometabolic conditions such as diabetes and hypertension, and oxidative stress [5,25,26,27].

Conversely, investigations of the relationship between CKD and PD have yielded conflicting results. A notable Korean cohort study previously found no significant correlation between these two conditions over a follow-up period of 9 years [28]. Additionally, thus far, only a few large-scale epidemiological examples of research have investigated the hazard of PD in patients with CKD to validate this issue [17,18,28]. However, in these studies, the sample sizes of the CKD groups and control groups were uneven in terms of demographic data. For example, the control groups were only matched to the CKD groups on the basis of age and sex [17,18], and socio-economic level was not accounted for in the analyses [18,28]. Furthermore, the CKD groups were generally older and exhibited a higher prevalence of comorbidities such as diabetes, hypertension, or hyperlipidemia [18,28]. Consequently, further validation employing national population cohort data with balanced demographics is necessary to mitigate the impact of confounding factors. Given that CKD and PD seem to exhibit mutual risk factors and possible reciprocal associations, it is of critical importance to conduct a longitudinal follow-up study, taking into account potential common confounders, to substantiate the link between CKD and the propensity toward developing PD.

In this study, we posited that the influence of CKD on the likelihood of developing PD may differ depending on patient-specific factors such as sex, age, socioeconomic status, and the presence of other comorbidities. The primary objective of this study was to scrutinize the incidence of PD and propose potential preventative strategies for individuals diagnosed with CKD. To accomplish this, we performed a longitudinal follow-up study to evaluate the association between CKD and the probability of PD development, utilizing data derived from the Korean national public healthcare system.

## 2. Materials and Methods

### 2.1. Ethics

This investigation was approved by the ethics committee of Hallym University (2019-10-023). The Institutional Review Board, following its guidelines and regulations, dispensed with the need for obtaining written informed consent. This research made use of the Korean National Health Insurance Service-Health Screening Cohort (KNHIS-HSC) data, a resource that offers anonymized, population-based digital records for research objectives, as previously described [29]. The diagnostic codes applied in this study adhered to the International Classification of Diseases, 10th Revision, Clinical Modification (ICD-10-CM) standards [30].

### 2.2. Exposure (CKD)

The researchers identified participants with CKD as those who had received at least two diagnoses of CKD (as per ICD-10 code: N18) or unspecified kidney failure (ICD-10 code: N19). Participants who had undergone routine dialysis treatment, including hemodialysis and/or peritoneal dialysis, were also included, provided that they had the corresponding treatment codes (O7010, O7020, and O7070).

### 2.3. Outcome (PD)

In the context of this research, PD was characterized using the ICD-10 code G20 (representing PD). To uphold the diagnostic precision, only participants who had undergone two or more clinical visits were incorporated into the analysis.

### 2.4. Participant Selection

Using the KNHIS-HSC dataset, individuals aged ≥40 years with medical claim codes between 2002 and 2019 were included, resulting in a total of 514,866 adult patients with 895,300,177 medical claim codes. Among these patients, 17,478 were identified as having CKD. Individuals who were not diagnosed with CKD between 2002 and 2019 were included in the control group (*n* = 497,388). However, to select first-time diagnosed CKD participants, those diagnosed in 2002 were omitted (*n* = 536) to allow a 1-year washout period. Additionally, CKD participants with missing records for BMI (*n* = 2), fasting blood glucose (*n* = 2), and blood pressure (*n* = 1) were excluded. Control participants identified with ICD-10 codes N18 once were also omitted (*n* = 560).

**Figure 1:**
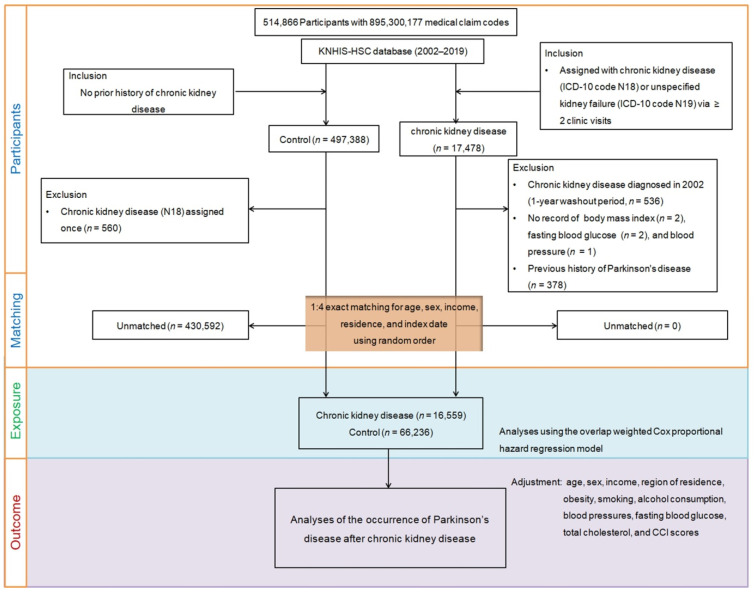
A schematic illustration of the participant selection process used in the present study. Of 514,866 participants, 16,559 participants with CKD were matched with 66,236 control participants based on age, sex, income, and region of residence. BMI, body mass index; CCI, Charlson Comorbidity Index; CKD, chronic kidney disease; DBP, diastolic blood pressure; ICD-10; International Classification of Diseases, 10th Revision; SBP, systolic blood pressure.

To reduce discrepancies in baseline demographic and clinical characteristics between CKD and control groups, propensity score matching was conducted. This matching process involved pairing participants with CKD with control participants with similar propensity scores based on age, sex, income, and place of residence. To guarantee impartial selection, the control group was randomized and chosen from the top of the list in a one-to-one match with CKD participants. Furthermore, to concurrently assess both groups, the index date for the control participants was set to be identical to their corresponding CKD participants. To ensure comparability between the CKD and control groups, any participant from the control group who died prior to the index date was discounted from the analysis. Additionally, in both groups, participants with a history of PD prior to the index date were discounted. In the CKD group, a total of 378 participants were discounted because of left truncation, implying that they did not meet the inclusion criteria during the matching procedure. Similarly, in the control group, 430,592 participants were eliminated during the matching procedure. Post-exclusion, 16,559 CKD participants were chosen and matched with 66,236 comparison group people in a 1:4 ratio. The participant selection and matching procedure is illustrated in Figure 1.

We endeavored to find newly established cases of PD by recognizing fresh assignments of ICD-10 codes for PD in both the CKD and comparison cohorts within the timeframe spanning from each individual’s index date up to the conclusion of the research period (2019).

### 2.5. Covariates

This study stratified participants into 10 age groups, each representing a 5-year age bracket, and 5 income brackets from class 1 (lowest income) to class 5 (highest income). The participants’ place of residence was classified as either urban or rural, following the methodology employed in a prior study [31]. We employed the same categorization protocol from this earlier study for three variables, including tobacco use, alcohol consumption, and obesity, which was determined based on the participant’s body mass index (BMI) measured in kg/m [31]. Obesity was classified into five categories using BMI (kg/m^2^) on the basis of the Asia-Pacific criteria and the Western Pacific Regional Office 2000 criteria as follows: underweight, <18.5, normal, 18.5–23, overweight, 23–25, obese I, 25–30, and obese II, ≥30 [32].

Health metrics, including systolic and diastolic blood pressure (mmHg) for hypertension status [33], fasting blood glucose levels (mg/dL) for hyperglycemia or diabetes status [34], and total cholesterol levels (mg/dL) for hyperlipidemia status [35], were also considered in this study. We used the Charlson Comorbidity Index (CCI) to evaluate the overall disease burden in the participants. The CCI considers the presence of 17 different comorbid conditions, assigning each participant a score based on the number and severity of diseases [36,37]. The CCI score can range from 0, indicating no comorbidities, to 29, signifying multiple comorbidities. In this analysis, CKD (represented by ICD-10 codes N18 and N19) was omitted from the CCI score, and the CCI was used as a continuous variable.

### 2.6. Statistical Analyses

Categorical data are presented as percentages, while continuous data are presented as means with their corresponding standard deviations. We used the standardized difference to compare the distribution of general attributes between the cohorts. Moreover, propensity score overlap weighting was utilized to ensure balanced covariates and amplify the effective sample size. The propensity score was computed via multivariable logistic regression, incorporating all covariates.

In the overlap-weighting scheme, CKD participants were weighted according to the propensity score’s probability, whereas control participants were weighted according to the probability of 1 minus the propensity score. We used this overlap weighting, ranging between 0 and 1, to achieve an ideal balance and enhance precision in the analyses [38,39,40].

This study utilized standardized differences to compare the general characteristics between the CKD and control groups, both before and after weighting. Additionally, the effectiveness of the matching process was assessed by comparing the absolute standardized differences of the covariates before and after matching. We regarded an absolute standardized difference of less than 0.20 as an indication of satisfactory balance [41].

Furthermore, the crude incidence rates and the differences in incidence rates were computed by dividing the number of participants who experienced a particular event by the total person-years of observation, and this was expressed as instances per 1000 person-years. We used the Kaplan–Meier method and the log-rank test to compare the cumulative incidence of PD in the CKD group with that in the control group.

To account for possible confounding factors and estimate the overlap-weighted hazard ratios (HRs) and 95% confidence intervals (CIs) for the incidence of PD in CKD patients, we utilized Cox proportional hazard regression models with overlap weighting. This was performed for both crude (unadjusted) and overlap-weighted (adjusted for factors such as age, sex, income, place of residence, obesity, smoking status, alcohol consumption, systolic blood pressure, diastolic blood pressure, fasting blood glucose levels, total cholesterol levels, and CCI scores) models.

The statistical analyses were performed using SAS software, version 9.4 (SAS Institute Inc., Cary, NC, USA). All analyses were two-tailed, and a *p*-value of less than 0.05 was considered to denote statistical significance.

## 3. Results

### 3.1. Baseline Characteristics

The study incorporated a cohort of 16,559 CKD patients who were age-, sex-, income-, and residence-matched with a control group of 66,236 individuals. The demographic and health-related characteristics of the participants at baseline, both before and after overlap-weighting adjustments for propensity-score matching, are presented in Table 1.

Before adjustment (crude), all covariates had a standardized difference of 0.00, indicating no disparities between the CKD and control groups with regard to age, sex, income, and region of residence. However, there were noticeable imbalances in certain baseline characteristics such as total cholesterol level, alcohol consumption, blood pressure readings, fasting blood glucose levels, smoking status, obesity status, and the CCI score. Upon employing overlap-weighting adjustments, these discrepancies were significantly reduced, achieving a standardized difference of less than 0.2 for all covariates. This suggests a balanced distribution of demographic and health-related attributes between the CKD and non-CKD groups post-adjustment.

### 3.2. The Occurrence of PD in the CKD and Control Groups

Before conducting the Cox analysis, we assessed the assumptions of the Cox model. The results showed that the *p*-value was 0.3973, indicating no violation of the Cox model assumption (Supplementary Table S1).

**Figure 2:**
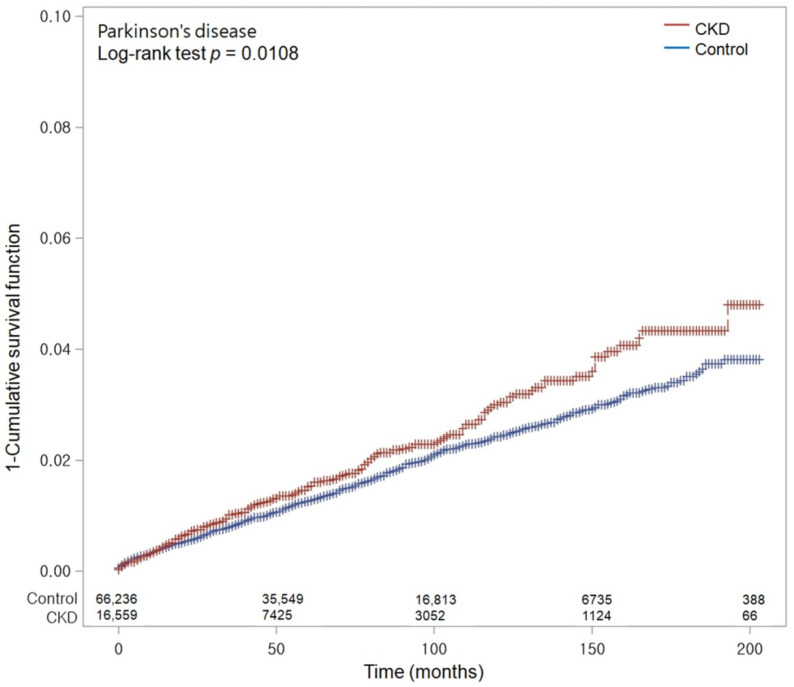
Kaplan–Meier probability of the incidence of Parkinson’s disease (PD) in chronic kidney disease (CKD) and control populations within 16 years of the index date.

Table 2 shows the raw and adjusted HRs for the incidence of PD in CKD patients. We found that the incidence rates of PD within the follow-up durations of 70,323 person-years for the CKD group and 343,256 person-years for the control group were 3.41 and 2.75 per 1000 person-years, respectively. Notably, the Kaplan–Meier analysis and log-rank test revealed a significantly elevated cumulative incidence of PD in the CKD group during the follow-up period compared to the control group (*p* = 0.0108; Figure 2).

In the unadjusted model, the HR for PD incidence was notably higher in the CKD group than in the control group (crude HR, 1.20; 95% CI = 1.04–1.39; *p* = 0.011). However, after adjusting for demographic characteristics and medical comorbidities using Cox regression analysis, the difference was not statistically significant (1.09; 95% CI = 0.97–1.22; *p* = 0.147). We conducted competing risk analysis to examine the association between CKD and PD in our study population. The results of the analysis indicated that both the crude and adjusted subdistribution HRs were 1.03 and 0.92, respectively (*p* = 0.726 and *p* = 0.428, respectively), indicating that the presence of CKD did not significantly impact the development of PD in our study population (Supplementary Table S2).

### 3.3. Subgroup Analysis

We conducted a deeper exploration of the relationship between CKD and the incidence of PD by segmenting the patients based on variables such as age, sex, income, place of residence, weight status, smoking and drinking habits, fasting blood glucose levels, total cholesterol levels, and CCI scores (Table 2 and Table 3). Notably, we observed the loss of statistical significance in the adjusted hazard ratios in several patient subgroups. This included younger individuals (aged <70), females, non-smokers, and those consuming alcohol less than once a week, although the raw hazard ratios showed a higher incidence rate of PD in these CKD subgroups (aged <70 years: 1.39, 95% CI = 1.09–1.78, *p* = 0.008; female: 1.27, 95% CI = 1.01–1.60, *p* = 0.039; non-smoker: 1.19, 95% CI = 1.00–1.40, *p* = 0.047; and alcohol consumption <1 time a week: 1.19, 95% CI = 1.02–1.40, *p* = 0.031).

**Figure 3:**
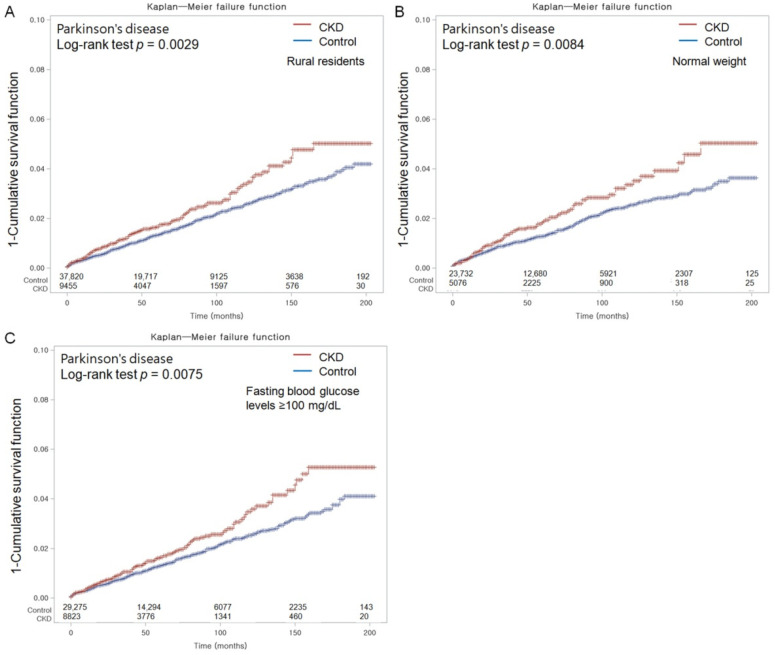
Kaplan–Meier probability of the incidence of Parkinson’s disease (PD) in chronic kidney disease (CKD) and control populations according to subgroups of rural residents (**A**), normal weight (**B**), and fasting blood glucose ≥ 100 mg/dL (**C**) within 16 years of the index date.

Nevertheless, certain subgroups of CKD patients exhibited a higher likelihood of PD development. These included those residing in rural areas (adjusted HR (aHR), 1.19; 95% CI = 1.03–1.37; *p* = 0.022), those with a normal weight (aHR, 1.29; 95% CI = 1.08–1.56; *p* = 0.006), and those with fasting blood glucose levels ≥100 mg/dL (aHR, 1.18; 95% CI = 1.00–1.39; *p* = 0.046). The Kaplan–Meier analyses with log-rank tests also revealed a significantly elevated cumulative incidence of PD in the CKD group during the follow-up period compared to the control group, in those subgroups of rural residents, normal weight, and fasting blood glucose levels ≥100 mg/dL (Figure 3).

## 4. Discussion

By leveraging propensity score overlap-weighted Cox proportional hazard regression analysis that considered confounding factors such as demographic, socioeconomic, lifestyle, and comorbidity variables, this study revealed that the overall CKD group may not be at an elevated risk of developing PD compared to the non-CKD group. However, the risk association exhibited variance contingent on specific lifestyle and comorbidity factors. In fact, an enhanced propensity towards PD was identified within particular subgroups of CKD patients, namely those residing in rural areas, individuals with a normal weight, and those with elevated blood glucose levels. This implies that the pathophysiological mechanisms contributing to the subsequent occurrence of PD in a subset of CKD patients are multifaceted, suggesting that lifestyle modifications might play a significant role in disease prevention strategies against potential PD development in CKD patients.

Our findings partially concur with those of another Korean population-based study, which reported no significant association between overall CKD and PD in CKD patients over a 9-year analysis period, even after adjusting for age, sex, and comorbidities such as high blood pressure, diabetes mellitus, and dyslipidemia [28]. This particular study also indicated a significantly elevated risk of incident PD in male patients with advanced CKD (HR, 3.71; 95% CI = 1.54–8.91) [28]. However, they included a relatively smaller sample of CKD patients (only 2998), and their methodology for selecting control subjects was unclear, with no matching for factors such as age, sex, income, or residence between CKD patients and controls [28].

In contrast, our results differ from those of another Korean study and two Taiwanese cohort studies [17,18,19]. While the Korean cohort study suggested a positive correlation between CKD severity and PD [19], its participant pool only comprised individuals aged ≥65 years [19], which may have introduced selection bias. One of the Taiwanese studies utilized a national insurance claims database (comprising 8325 CKD patients and a control cohort of 33,382) and reported a significant 1.73-fold greater risk (95% CI = 1.39–2.15) of PD associated with CKD or end-stage renal disease, especially in female and younger CKD patients, compared to the control cohort over a 2.56-year follow-up [17]. Additionally, the other Taiwanese cohort study (including 2862 chronic renal failure patients and a control group of 14,310) found that after adjusting for diabetes mellitus over a 3-year follow-up, individuals with chronic renal failure had a 1.81-fold bigger risk (95% CI = 1.21–2.71) of developing parkinsonism when compared to the comparison group; however, this study did not primarily focus on PD patients, and the 3-year follow-up period may have been insufficient for the comprehensive assessment of PD development.

While previous studies have identified a high prevalence of CKD and PD among certain demographic groups, including men [28], women [17], and the elderly [19], our study achieved a balanced distribution of demographic and health-related factors by matching 16,559 individuals with CKD to 66,236 participants without CKD. This approach enabled us to accurately explore the relationship between CKD and PD [39]. In doing so, we determined that while the overall CKD group may not be at higher risk of PD in comparison with the non-CKD group, a subset of the CKD group under certain conditions (such as rural residents, individuals with a normal weight, and those with hyperglycemia) showed an elevated likelihood of developing PD during the 16-year long-term follow-up analysis.

In the current investigation, an association was observed between CKD and PD in the crude model; however, this relevance was no longer statistically significant after adjustments were made for sociodemographic and comorbidity factors such as age, sex, income, residential place, obesity status, smoking habits, alcohol drinking, systolic and diastolic blood pressure, fasting blood glucose levels, total cholesterol, and CCI scores. This adjusted analysis could imply that these clinical and environmental parameters potentially mediate the incidence of PD in CKD individuals, considering the consensus that both genetic and environmental factors contribute to the development of PD [25].

Several elements, including uremic toxins, oxidative stress, and chronic inflammation, have been highlighted as noteworthy factors in the onset of age-related neurodegenerative disorders such as PD among individuals with CKD [27,42]. Due to the closely intertwined anatomical and physiological aspects, the kidney–brain axis communicates to maintain body homeostasis [21,22]. This kidney–brain interaction plays an essential role in the pathophysiology of neurological conditions, a connection that has only recently gained recognition [43]. Interestingly, all studies addressing the association between CKD and PD were performed in Asia, predominantly in Taiwan and Korea [17,18,19,28]. Therefore, further research is required to investigate whether this relevance is evident in other ethnicities.

Furthermore, the mechanisms underlying the association of a subset of CKD patients with the development of PD, particularly in rural residents, individuals of normal weight, and those with hyperglycemia, remain elusive. A previous study has suggested that a reduced glomerular filtration rate could independently contribute to an augmented hazard of PD development in patients with type 2 diabetes, thereby underlining the critical role of diabetic CKD in PD progression [44]. Interestingly, similar disruptions in glucose and energy metabolism that take place in type 2 diabetes are also observed in the early stages of sporadic PD [45]. Moreover, it is known that insulin receptors, primarily located in the basal ganglia and substantia nigra, are crucial for maintaining neuronal survival and growth, dopaminergic transmission, and synaptic integrity [46].

It has also been reported that individuals with CKD who have a normal weight exhibit a higher propensity for conditions such as cardiovascular disease and diabetes, as well as increased insulin resistance and a heightened risk of mortality [47,48]. Similarly, one case–control study suggested that a low BMI could potentially increase the risk of PD incidence [49]. In fact, among new PD patients (*n* = 398), a low BMI has been linked with a reduced density of nigrostriatal dopaminergic neurons [48], which provides further support to the notion that a low body weight contributes to PD-related pathologies [48].

Concerning geographical location, a Canadian cross-sectional study reported that CKD was more prevalent in rural areas (86.2 per 1000) than in urban areas (68.4 per 1000) [50]. This study also noted a higher prevalence of CKD among individuals with comorbid PD (223.7 per 1000), dementia (303.3 per 1000), and a combination of both diabetes and hypertension (267.4 per 1000) [50], suggesting a potential kidney–brain axis.

In addition, there are genetic causes of PD, with the most common being mutations in leucine-rich repeat kinase 2 (*LRRK2*) [9]. The LRRK2 protein is ubiquitously expressed, with the highest levels observed in the kidneys, lungs, and brain [51]. Notably, a previous preclinical toxicology study indicated a possible kidney pathology resulting from various LRRK2 inhibitors [52]. This suggests that *LRRK2* mutations could influence physiological processes and play disease-relevant roles beyond the nervous system [51].

The robustness and credibility of this research are strengthened by the use of a representative national cohort database, which facilitated the pairing of patients and control subjects through overlap-weighted propensity score matching. This methodology minimized selection bias and created study groups analogous to those seen in randomized clinical trials, thereby bolstering the validity of the study [39]. Additionally, the employment of the KNHIS-HSC database in our investigation ensured comprehensive access to each participant’s medical history from hospitals and clinics nationwide, enhancing the generalizability and precision of our results. A further strength of our study was the careful consideration and adjustment for potential confounding variables, encompassing socioeconomic factors such as income and place of residence; lifestyle-related risk factors such as alcohol consumption, blood pressure, obesity, fasting blood glucose, total cholesterol level, and smoking; and existing comorbidities. This comprehensive adjustment process augmented the reliability and accuracy of our findings. Lastly, our study’s 16-year follow-up period, one of the longest in examining the association between CKD and PD, provided a significant advantage in detecting and scrutinizing potential relationships between these two conditions over a prolonged timespan.

However, our study had some limitations. First, due to its observational and retrospective design, it is not possible to firmly establish a causal link between CKD and PD. Furthermore, we did not explore the underlying mechanisms that could clarify the correlation between these two conditions. Second, our study focused solely on Korean citizens aged over 40 years, relying on diagnosis codes from Korean health insurance data. This may have led to unmeasured confounding variables not being accounted for, which limits the extrapolation of our results to other demographic groups. Third, the KNHIS-HSC database did not contain details regarding the severity of CKD or PD, familial medical history, genetic factors, or dietary preferences. Additionally, essential clinical markers such as creatinine, glomerular filtration rate, or information on urate lowering therapies, diuretics, or drugs associated with PD were not provided in this study. This may have hindered our capacity to comprehensively understand and analyze the association between CKD and PD.

## 5. Conclusions

This comprehensive, nationwide, population-based study indicates that while the overall CKD population in Korea may not exhibit an elevated risk for PD when compared to the non-CKD population, particular subsets of CKD patients—including rural residents, those with a normal weight, and those with hyperglycemia—exhibit an increased likelihood of developing PD. These findings emphasize the need for targeted information and education regarding potential PD risks for CKD patients that fall into these categories. Therefore, the implementation of regular PD screening protocols and the encouragement of lifestyle adjustments may prove beneficial in preventing the potential onset of PD in these specific CKD patient subgroups. By recognizing certain lifestyle factors correlated with a heightened PD risk, CKD patients may be able to alter their behavior patterns to minimize or evade future PD susceptibility. However, further research is warranted to corroborate these associations and investigate the underlying mechanisms.

## Figures and Tables

**Table 1:** General characteristics of the participants.

Characteristics	Before Overlap-Weighting Adjustment (Crude)			After Overlap-Weighting Adjustment		
	CKD (*n* = 16,559)	Control (*n* = 66,236)	Standardized Difference	CKD (*n* = 16,559)	Control (*n* = 66,236)	Standardized Difference
Age (y) (%)			0.00			0.00
40–44	0.59	0.59		0.61	0.61	
45–49	2.19	2.19		2.14	2.14	
50–54	5.72	5.72		5.60	5.60	
55–59	11.24	11.24		11.10	11.10	
60–64	13.86	13.86		13.65	13.65	
65–69	15.53	15.53		15.42	15.42	
70–74	17.53	17.53		17.60	17.60	
75–79	16.92	16.92		17.11	17.11	
80–84	11.04	11.04		11.21	11.21	
≥85	5.37	5.37		5.57	5.57	
Sex (%)			0.00			0.00
Male	65.89	65.89		65.97	65.97	
Female	34.11	34.11		34.03	34.03	
Income (%)			0.00			0.00
1 (lowest)	17.41	17.41		17.32	17.32	
2	11.49	11.49		11.52	11.52	
3	14.27	14.27		14.23	14.23	
4	19.95	19.95		19.87	19.87	
5 (highest)	36.88	36.88		37.07	37.07	
Region of residence (%)			0.00			0.00
Urban	42.90	42.90		42.92	42.92	
Rural	57.10	57.10		57.08	57.08	
Obesity (%)			0.17			0.00
Underweight	2.63	3.43		2.80	2.80	
Normal	30.65	35.83		31.67	31.67	
Overweight	26.19	27.05		26.50	26.50	
Obese I	35.94	30.96		35.03	35.03	
Obese II	4.58	2.73		4.00	4.00	
Smoking status (%)			0.02			0.00
Nonsmoker	63.72	64.68		64.02	64.02	
Past smoker	10.51	10.65		10.62	10.62	
Current smoker	25.77	24.67		25.36	25.36	
Alcohol consumption (%)			0.07			0.00
<1 time a week	72.55	69.58		71.80	71.80	
≥1 time a week	27.45	30.42		28.20	28.20	
SBP, mean (SD)	131.83 (18.36)	128.80 (16.36)	0.18	130.93 (15.43)	130.93 (7.34)	0.00
DBP, mean (SD)	78.76 (11.51)	78.10 (10.39)	0.06	78.55 (9.82)	78.55 (4.56)	0.00
Fasting blood glucose, mean (SD)	115.57 (49.20)	103.51 (28.33)	0.30	109.87 (32.58)	109.87 (17.36)	0.00
Total cholesterol, mean (SD)	190.38 (45.70)	193.18 (38.62)	0.07	190.76 (39.08)	190.76 (16.94)	0.00
CCI score, mean (SD)	2.16 (2.19)	1.13 (1.72)	0.53	1.82 (1.68)	1.82 (0.98)	0.00
Parkinson’s disease (%)	1.45	1.43	0.00	1.37	1.59	0.02

Abbreviations: CCI, Charlson Comorbidity Index; SBP, systolic blood pressure; DBP, diastolic blood pressure; CKD, chronic kidney disease; SD, standard deviation.

**Table 2:** Crude and propensity score overlap-weighted HRs and 95% CIs of CKD for PD, with subgroup analyses according to age, sex, income, and region of residence.

	N of Event/ N of Total (%)	Follow-Up Duration (PY)	IR per 1000 (PY)	IRD (95% CI)	HRs for PD			
					Crude	*p*	Overlap-Weighted Model ^†^	*p*
Total participants								
CKD	240/16,559 (1.45)	70,323	3.41	0.66 (0.23–1.09)	1.20 (1.04–1.39)	0.011 *	1.09 (0.97–1.22)	0.147
Control	945/66,236 (1.43)	343,256	2.75		1		1	
Aged <70 years								
CKD	83/8137 (1.02)	45,642	1.82	0.49 (0.11–0.87)	1.39 (1.09–1.78)	0.008 *	1.14 (0.94–1.39)	0.188
Control	287/32,548 (0.88)	216,298	1.33		1		1	
Aged ≥70 years								
CKD	157/8422 (1.86)	24,681	6.36	1.18 (0.18–2.18)	1.17 (0.98–1.39)	0.082	1.05 (0.92–1.21)	0.472
Control	658/33,688 (1.95)	126,958	5.18		1		1	
Male								
CKD	146/10,911 (1.34)	45,283	3.22	0.52 (−0.01–1.06)	1.16 (0.97–1.39)	0.106	1.10 (0.95–1.27)	0.211
Control	595/43,644 (1.36)	220,572	2.70		1		1	
Female								
CKD	94/5648 (1.66)	25,040	3.75	0.90 (0.16–1.65)	1.27 (1.01–1.60)	0.039 *	1.05 (0.88–1.26)	0.598
Control	350/22,592 (1.55)	122,684	2.85		1		1	
Low-income group								
CKD	91/7148 (1.27)	30,067	3.03	0.61 (−0.01–1.23)	1.22 (0.97–1.54)	0.091	1.04 (0.86–1.25)	0.716
Control	361/28,592 (1.26)	149,326	2.42		1		1	
High-income group								
CKD	149/9411 (1.58)	40,256	3.70	0.69 (0.09–1.29)	1.19 (0.99–1.43)	0.058	1.12 (0.97–1.29)	0.12
Control	584/37,644 (1.55)	193,930	3.01		1		1	
Urban resident								
CKD	89/7104 (1.25)	32,137	2.77	0.23 (−0.39–0.84)	1.05 (0.84–1.33)	0.652	0.96 (0.80–1.15)	0.63
Control	390/28,416 (1.37)	153,263	2.54		1		1	
Rural resident								
CKD	151/9455 (1.60)	38,186	3.95	1.03 (0.42–1.64)	1.31 (1.10–1.57)	0.003 *	1.19 (1.03–1.37)	0.022 *
Control	555/37,820 (1.47)	189,993	2.92		1		1	

Abbreviation: CKD, chronic kidney disease; PD, Parkinson’s disease; IR, incidence rate; IRD, incidence rate difference; PY, person-year; HR, hazard ratio; CI, confidence interval. * Significance at *p* < 0.05. ^†^ Adjusted for age, sex, income, region of residence, obesity, smoking, alcohol consumption, systolic blood pressure, diastolic blood pressure, fasting blood glucose, total cholesterol, and Charlson Comorbidity Index scores.

**Table 3:** Subgroup analyses of the crude and propensity score overlap-weighted hazard ratios and 95% confidence intervals of CKD for PD.

	N of Event/ N of Total (%)	Follow-Up Duration (PY)	IR per 1000 (PY)	IRD (95% CI)	Hazard Ratios for PD			
					Crude	*p*	Overlap-Weighted Model ^†^	*p*
Underweight								
CKD	7/436 (1.61)	1334	5.25	2.29 (−0.97–5.54)	1.68 (0.73–3.83)	0.22	1.78 (0.91–3.46)	0.091
Control	29/2275 (1.27)	9794	2.96		1		1	
Normal weight								
CKD	86/5076 (1.69)	20,826	4.13	1.24 (0.43–2.06)	1.37 (1.08–1.74)	0.009 *	1.29 (1.08–1.56)	0.006 *
Control	351/23,732 (1.48)	121,552	2.89		1		1	
Overweight								
CKD	58/4336 (1.34)	19,479	2.98	0.43 (−0.36–1.22)	1.15 (0.86–1.53)	0.341	0.98 (0.78–1.23)	0.891
Control	244/17,917 (1.36)	95,620	2.55		1		1	
Obese								
CKD	89/6711 (1.33)	28,684	3.10	0.34 (−0.34–1.03)	1.09 (0.86–1.38)	0.468	0.95 (0.78–1.15)	0.583
Control	321/22,312 (1.44)	116,290	2.76		1		1	
Non-smoker								
CKD	170/10,552 (1.61)	46,431	3.66	0.66 (0.10–1.22)	1.19 (1.00–1.40)	0.047 *	1.07 (0.94–1.22)	0.324
Control	677/42,843 (1.58)	225,488	3.00		1		1	
Past and current smoker								
CKD	70/6007 (1.17)	23,892	2.93	0.65 (−0.03–1.33)	1.24 (0.95–1.62)	0.108	1.12 (0.91–1.39)	0.293
Control	268/23,393 (1.15)	117,768	2.28		1		1	
Alcohol consumption <1 time a week								
CKD	187/12,014 (1.56)	52,009	3.60	0.68 (0.16–1.20)	1.19 (1.02–1.40)	0.031 *	1.08 (0.94–1.23)	0.269
Control	701/46,084 (1.52)	240,457	2.92		1		1	
Alcohol consumption ≥1 time a week								
CKD	53/4545 (1.17)	18,314	2.89	0.52 (−0.26–1.30)	1.19 (0.88–1.60)	0.252	1.13 (0.90–1.42)	0.295
Control	244/20,152 (1.21)	102,799	2.37		1		1	
SBP < 140 mmHg and DBP < 90 mmHg								
CKD	142/10,873 (1.31)	44,452	3.19	0.54 (0.02–1.07)	1.17 (0.97–1.40)	0.098	1.05 (0.91–1.21)	0.496
Control	642/48,027 (1.34)	242,312	2.65		1		1	
SBP ≥ 140 mmHg or DBP ≥ 90 mmHg								
CKD	98/5686 (1.72)	25,871	3.79	0.79 (0.02–1.55)	1.23 (0.98–1.54)	0.079	1.13 (0.93–1.38)	0.21
Control	303/18,209 (1.66)	100,944	3.00		1		1	
Fasting blood glucose <100 mg/dL								
CKD	105/7736 (1.36)	35,665	2.94	0.30 (−0.28–0.88)	1.08 (0.88–1.33)	0.467	1.00 (0.86–1.17)	0.963
Control	547/36,961 (1.48)	206,922	2.64		1		1	
Fasting blood glucose ≥100 mg/dL								
CKD	135/8823 (1.53)	34,658	3.90	0.98 (0.32–1.63)	1.30 (1.07–1.59)	0.008 *	1.18 (1.00–1.39)	0.046 *
Control	398/29,275 (1.36)	136,334	2.92		1		1	
Total cholesterol <200 mg/dL								
CKD	148/10,248 (1.44)	40,352	3.67	0.68 (0.08–1.28)	1.18 (0.99–1.41)	0.072	1.05 (0.91–1.22)	0.513
Control	574/38,964 (1.47)	192,099	2.99		1		1	
Total cholesterol ≥200 mg/dL								
CKD	92/6311 (1.46)	29,971	3.07	0.62 (−0.01–1.24)	1.23 (0.98–1.54)	0.08	1.15 (0.96–1.38)	0.123
Control	371/27,272 (1.36)	151,157	2.45		1		1	
CCI score = 0								
CKD	39/4900 (0.80)	23,012	1.69	0.30 (−0.21–0.82)	1.19 (0.85–1.67)	0.306	1.24 (0.99–1.56)	0.061
Control	265/35,464 (0.75)	190,299	1.39		1		1	
CCI score = 1								
CKD	33/2828 (1.17)	11,519	2.86	−0.90 (−2.09–0.30)	0.75 (0.52–1.07)	0.114	0.81 (0.62–1.05)	0.113
Control	239/12,315 (1.94)	63,533	3.76		1		1	
CCI score ≥ 2								
CKD	168/8831 (1.90)	35,792	4.69	−0.24 (−1.09–0.62)	0.92 (0.77–1.10)	0.377	1.03 (0.87–1.22)	0.703
Control	441/18,457 (2.39)	89,424	4.93		1		1	

Abbreviations: CKD, chronic kidney disease; PD, Parkinson’s disease; IR, incidence rate; IRD, incidence rate difference; PY, person-year; CI, confidence interval; CCI, Charlson Comorbidity Index; SBP, systolic blood pressure; DBP, diastolic blood pressure. * Significance at *p* < 0.05. ^†^ Adjusted for age, sex, income, region of residence, obesity, smoking, alcohol consumption, systolic blood pressure, diastolic blood pressure, fasting blood glucose, total cholesterol, and Charlson Comorbidity Index scores.

## Data Availability

All data are available from the National Health Insurance Sharing Service (NHISS) database (https://nhiss.nhis.or.kr; accessed on 1 July 2022). The NHISS allows access to all these data for any researcher who promises to follow the research ethics, at some processing charge. If you wish to access the data of this article, you can download it from the website after promising to follow the research ethics.

## Supplementary Materials

The following supporting information can be downloaded at: https://www.mdpi.com/article/10.3390/nu15143205/s1, Supplementary Table S1; *p*-value of zph-test between chronic kidney disease and Parkinson’s disease. Supplementary Table S2; Fine and Gray regression analysis in Parkinson’s disease between chronic kidney disease and control groups.
