# Supplementary material for: Exploring the Link between Chronic Kidney Disease and Parkinson’s Disease: Insights from a Longitudinal Study Using a National Health Screening Cohort
Source: Nutrients. 2023 Jul 19;15(14):3205. doi: 10.3390/nu15143205 (PMC10385674; doi:10.3390/nu15143205)
Supplement: Supplementary file 1 [file nutrients-15-03205-s001.zip › nutrients-2448331-supplementary.pdf]

Table S1. P-value of zph-test between chronic kidney disease and Parkinson's disease.

Characteristics	P-value
Parkinson's disease	0.3973

Table S2. Fine and Gray regression analysis in Parkinson's disease between chronic kidney disease and control groups.

	Subdistribution Hazard ratios (95% confidence interval)			
	Crude	P-value	Overlap weighted model †	P-value
Total participants				
CKD	1.03 (0.89–1.18)	0.726	0.92 (0.75–1.13)	0.428
Control	1		1	

† Adjusted for age, sex, income, region of residence, obesity, smoking, alcohol consumption, systolic blood pressure, diastolic blood pressure, fasting blood glucose, total cholesterol, and Charlson Comorbidity Index scores
